# In‐vitro spermatogenesis through testis modelling: Toward the generation of testicular organoids

**DOI:** 10.1111/andr.12741

**Published:** 2020-01-09

**Authors:** Guillaume Richer, Yoni Baert, Ellen Goossens

**Affiliations:** ^1^ Biology of the Testis research Lab Department of Reproduction, Genetics and Regenerative Medicine Vrije Universiteit Brussel (VUB) Brussels Belgium

**Keywords:** in‐vitro spermatogenesis, spermatogonial stem cell niche, testicular organoid, testicular scaffold, tubulogenesis

## Abstract

**Background:**

The testicular organoid concept has recently been introduced in tissue engineering to refer to testicular cell organizations modeling testicular architecture and function. The testicular organoid approach gives control over which and how cells reaggregate, which is not possible in organotypic cultures, thereby extending the applicability of in‐vitro spermatogenesis (IVS) systems. However, it remains unclear which culture method and medium allow reassociation of testicular cells into a functional testicular surrogate in‐vitro.

**Objective:**

The aim of this paper is to review the different strategies that have been used in an attempt to create testicular organoids and generate spermatozoa. We want to provide an up‐to‐date list on culture methodologies and media compositions that have been used and determine their role in regulating tubulogenesis and differentiation of testicular cells.

**Search method:**

A literature search was conducted in PubMed, Web of Science, and Scopus to select studies reporting the reorganization of testicular cell suspensions in‐vitro, using the keywords: three‐dimensional culture, in‐vitro spermatogenesis, testicular organoid, testicular scaffold, and tubulogenesis. Papers published before the August 1, 2019, were selected.

**Outcome:**

Only a limited number of studies have concentrated on recreating the testicular architecture in‐vitro. While some advances have been made in the testicular organoid research in terms of cellular reorganization, none of the described culture systems is adequate for the reproduction of both the testicular architecture and IVS.

**Conclusion:**

Further improvements in culture methodology and medium composition have to be made before being able to provide both testicular tubulogenesis and spermatogenesis in‐vitro.

## 1 INTRODUCTION

Globally, the prevalence of male infertility has been estimated to range between 9% and 16%.^1^ Additionally, sperm counts among western populations have more than halved (52.4%) in the past 40 years and keep on decreasing by an average of 1.4% every year.^1^ This decrease in sperm count not only relates to fertilization rates but also represents a social and biological crisis.^2^ The causes for the catastrophic drop in sperm count are currently unknown. Also, our knowledge on testicular andrology and regulation of spermatogenesis is not complete, hindering research on male infertility, its diagnosis, and treatment.^3^ A culture system for the production of haploid spermatozoa starting from testicular cell suspensions would be a powerful tool to study testicular function through gain‐ or loss‐of‐function experiments. Additionally, the human culture system for in‐vitro spermatogenesis (IVS) could serve as an alternative to animal usage in high‐throughput screening assays in the industry. For instance, the European Registration, Evaluation, Authorisation and Restriction of Chemicals regulation (No. 1907/2006) has required toxicity testing for many chemicals on the market. These so‐called reprotoxicity tests have been estimated to cost at least €6.7 billion and involve 48.6 million animals.^4^ Moreover, prepubertal boys needing to undergo gonadotoxic treatments, and thus being at risk for spermatogonial stem cell (SSC) depletion, will highly benefit from in‐vitro derived spermatozoa because they are too young for sperm banking. Cryopreservation of testicular tissue is currently the only experimental procedure to preserve their reproductive function.^5^ Autografting of the stored testicular tissue or SSC transplantation is promising fertility preservation strategies. But, the risks related to the transplantation of residual malignant cells limit their application. In‐vitro maturation of cryostored SSCs into haploid spermatozoa for later usage in artificial reproduction would bring a promising fertility preservation option for childhood cancer survivors. Similarly, it would benefit the infertility treatment of the wide range of non‐obstructive azoospermia patients who are not able to produce spermatozoa but still have SSCs.

Germ cell differentiation relies on the support of Sertoli cells in the tubular compartment of the testis and on factors secreted from the peritubular myoid cells (PTMCs) and other interstitial cells, such as Leydig cells, endothelial cells, fibroblasts, and macrophages. Three types of three‐dimensional (3D) IVS culture systems have been successful in rodents: supportive matrix culture systems for testicular cell suspensions,^6^ organotypic culture of small testicular fragments^7^ and, recently, 3D bioprinting of testicular cell suspensions.^8^ Of these, only mouse organotypic culture systems resulted in offspring.^9^, ^10^, ^11^ Up to now, organotypic cultures offer the highest IVS efficiency by preserving the testicular architecture and maintaining complex cellular interactions. The translation of the organotypic culture to humans is still ongoing,^12^, ^13^^,^ 14 Although promising steps have been made, haploid cells were rarely found and the possible presence of residual differentiating germ cells in the starting material used in these approaches could have biased the evaluation of the results. Also, solid characterization of the in‐vitro derived germ cells is still needed.^15^ Moreover, this system does not allow researchers to manipulate specific cells before culture, to help understand the many mechanisms controlling testicular physiology and spermatogenesis, and to discover new clinical targets.

Culturing testicular cell suspensions may be a suitable alternative. The testicular organoid concept refers to the reconstitution of a functional in‐vitro environment from testicular cell suspensions, compatible with IVS.^16^, ^17^, ^18^, ^19^, ^20^ Recapitulation of specific aspects of testicular development and differentiation in‐vitro depends on the degree of physical support and the composition of the culture medium, but also on the cell types within the organoid itself. The aim of this review is to identify extrinsic factors driving testicular development from testicular cell suspensions, while supporting SSC differentiation in‐vitro.

## 2 SEARCH STRATEGY

A literature search was conducted in PubMed, Web of Science, and Scopus with combinations of the following research terms: three‐dimensional culture, in‐vitro spermatogenesis, testicular organoid, testicular scaffold, and tubulogenesis. To prevent recent articles to be missed, the search strategy was repeated weekly until August 1, 2019. Only studies reporting the reorganization of testicular cell suspensions in‐vitro were included with no restrictions to year of publication. Studies using embryonic stem cells, induced pluripotent stem cells, or grafting were excluded. Reference lists of the selected articles were screened for additional relevant studies. Data concerning culture method and culture medium composition were extracted from these articles and divided into three (2D, 3D, and novel 3D strategies) and six groups (basal medium, serum, gonadotrophins, TGFβ‐superfamily, other growth factors, and vitamin A), respectively. Because of a limited amount of human studies, rodent studies were also included. Moreover, organotypic culture studies were sometimes discussed to support the findings. Tables S1 and S2 provide detailed data derived from the selected articles.

## 3 IMPACT OF THE CULTURE METHOD ON TESTICULAR TUBULOGENESIS IN‐VITRO

In this section, the various culture methods that have been used in the past to study tubulogenesis in‐vitro starting from testicular cell suspensions are summarized. In recent years, a shift from conventional 2D (Table S1) toward 3D (Table S2) culture models has been observed, because of the latter's ability to replicate the extracellular‐matrix (ECM) features (eg, regulation of adhesion, migration, differentiation, and morphogenesis of embedded testicular cells) and the generation of the complex multi‐cellular organization of organs. Accordingly, novel 3D strategies and bio‐printers have recently been introduced to facilitate the establishment of the different testicular compartments in‐vitro.

### 3.1 Two‐dimensional cultures have limited potential in testicular tubulogenesis

**Figure 1:**
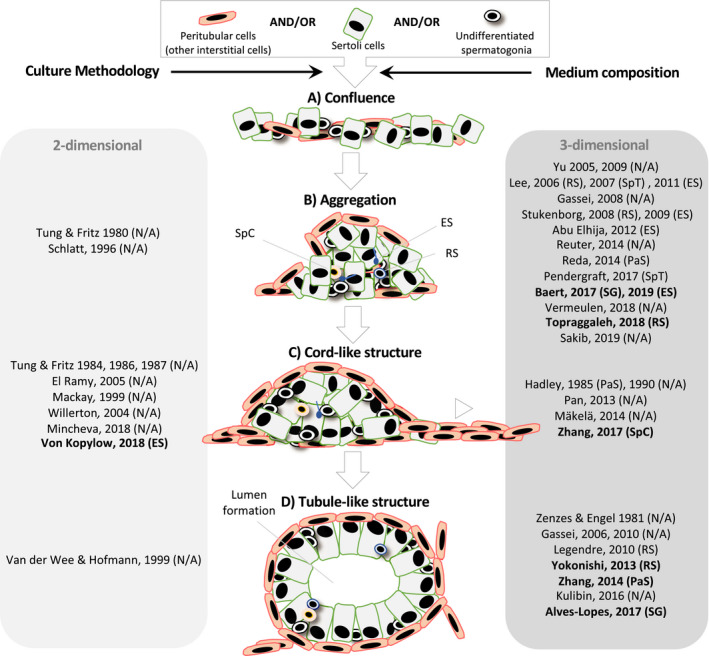
Structural reorganization of all or combinations of testicular cell types in‐vitro in chronological order. Testicular tubulogenesis in‐vitro comprises distinct phases: gaining of cell confluence (A). Aggregation of Sertoli cells into multinodular mounds under influence of contractile peritubular myoid cells (B). Interconnection and merging of multinodular mounds to form cable‐like structures (C). Formation of hollow tubules (D). A shift from 2D (light gray table) toward 3D (dark gray table) culture models has been observed because of the latter's ability to improve the cell reorganization. Aside from the culture methodology, medium composition influences the different aspects of testicular tubulogenesis in‐vitro. Of the different media‐ingredients, KSR has been proven critical (bold). Upon reorganization of the testicular cells, differentiation of spermatogonia could be seen. The most advanced differentiation stage in each study was indicated between parentheses. ES, elongates spermatids; PaS, pachytene spermatocytes; RS, round spermatids; SG, spermatogonia; SpC, spermatocytes; SpT, spermatids

The initial steps of testicular tubulogenesis have been described in 2D mono‐cultures of embryonic mouse Sertoli cells,^21^, ^22^ neonatal rat Sertoli cells,^23^ or immortalized mouse Sertoli cells,^24^ but also in 2D cultures containing all testicular cell types of neonatal rat,^25^, ^26^, ^27^, ^28^, ^29^, ^30^ or adult human.^31^^,^ 32 Following plating, testicular cells spread to form mono‐layered plaques (Figure 1A). The contractile activity of PTMCs reassembles Sertoli cells into mono‐layered islands. Subsequently, PTMCs elicit compaction and reaggregation of Sertoli cells into multinodular mounds (Figure 1B) that eventually merge through cytoplasmic projections (protrusions) to form cord‐like structures (Figure 1C). Interactions between Sertoli cells and PTMCs result in the deposition of basement membrane components that will separate the tubular (containing undifferentiated Sertoli cells and germ cells) from the surrounding interstitial compartment (containing PTMCs, among others). In response to signals from the basement membrane and interstitium, Sertoli cells within the aggregates differentiate into functional columnar‐shaped epithelial cells and establish an apical‐basal polarization by becoming perpendicular to the basement membrane upon differentiation (Figure 1D).

The importance of the basement membrane in testicular cord formation was evidenced by culturing rodent testicular cells in 2D cultures with different ECM coatings, such as laminin,^21^, ^24^, ^26^, ^28^, ^30^ fibronectin,^21^, ^24^, ^26^ collagen,^23^, ^24^, ^26^ seminiferous tubule biomatrix,^23^ and Matrigel^®^.^21^, ^22^, ^24^, ^33^ Neonatal rat Sertoli cells cultured on seminiferous tubule biomatrix and Matrigel^®^ reoriented into polarized cells with formation of tight junctions, more closely resembling their in‐vivo counterparts than when cultured on plastic surfaces. Moreover, the cells showed better functionality as measured by the secretion of collagen, laminin and fibronectin,^23^ and androgen‐binding protein.^33^ Similarly, enhanced cord‐like structures were reported when embryonic mouse Sertoli cells were grown on Matrigel^®^ 21, 22 Of the proteins present in these matrices, fibronectin guided the migration of Sertoli cells,^27^, ^34^ while laminin strongly stimulated the reorganization of Sertoli cells into cord‐like structures.^24^, ^28^ Nevertheless, only some of the formed cord‐like structures in rodent 2D cultures developed into tubule‐like structures with formation of a lumen.^24^ Noteworthy, Van der Wee and colleagues did not start from primary testicular cells, but used an immortalized Sertoli cell line.^24^ In studies reporting reorganization of adult human testicular cells, cord‐like structures could be formed when grown on glass or plastic.^31^, ^32^ However, survival of germ cells within the 2D‐derived structures was low^32^ and the effect of Matrigel^®^ was not evaluated. Altogether, the structures rarely established an organization comparable to that observed in‐vivo.^30^, ^31^, ^32^

### 3.2 Three‐dimensional cultures improve multilayered testicular cell reorganization

Testicular cells cultured within, rather than onto Matrigel^®^, reorganized faster and formed improved cord structures displaying better germ cell viability.^33^, ^35^ Interestingly, some spermatogonia surrounded by Sertoli cells inside the cord‐like structures differentiated up to late pachytene spermatocytes.^33^ These results suggest that 3D culture of testicular cells benefits over 2D culture by providing a more physiologically stable system allowing reorganization of embedded testicular cells in‐vitro with the potential to improve both tubulogenesis and spermatogenesis. In addition, Hadley et al^35^ emphasized the role of laminin as an important factor within Matrigel^®^ driving the reorganization of Sertoli cells into cords. Indeed, laminin is crucial for Sertoli and germ cell survival and differentiation through integrin‐dependent signaling.^36^ Matrigel^®^ has been used extensively for the embedding of neonatal rat^17^, ^30^, ^37^, ^38^, ^39^, ^40^, ^41^, ^42^ and mouse testicular cells.^19^ More post‐meiotic germ cells could be observed in neonatal rat testicular cell‐derived aggregates within a mixture of Matrigel^®^ and collagen gel, than in collagen gel alone.^40^ In accordance with Hadley and colleagues, the authors suggested that laminin was responsible for this effect. Interestingly, culturing neonatal rat testicular cells with an overlay of Matrigel^®^ promoted the multilayered aggregation of testicular cells, while improving survival of spermatogonia.^37^ These models have later been used to evaluate the toxic effects of certain chemicals, such as bisphenol A,^42^ cadmium^43^ and phthalates,^44^, ^45^, ^46^, ^47^ Likewise, Legendre et al^41^ developed an in‐vitro model of the blood‐testis barrier for toxicology studies. In their model, neonatal rat PTMCs were cultured on the basal side of a transwell bicameral culture chamber, while Sertoli cells and germ cells reorganized into tubule‐like structures within Matrigel^®^ at the apical side of the transwell.^41^ In their study, round spermatids could be detected. A recent study showed the ability of neonatal rat testicular cells to rebuild tubule‐like structures when resuspended in a drop of Matrigel^®^, placed between two other cell‐free Matrigel^®^ layers.^17^ In contrast to the conventional single‐layer approach, testicular cells organized into tubule‐like structures with a blood‐testis barrier, maintaining the mitotic activity of undifferentiated spermatogonia. However, because Matrigel^®^ is a mixture of not completely identified ECM proteins (mainly laminin, collagen IV, heparin sulfate proteoglycans, and entactin) and growth factors secreted by a mouse sarcoma, its use in clinical applications is limited.^48^

Collagen has been used to some extent for neonatal mouse^49^, ^50^, ^51^ and adult human testicular cells.^52^ Lee and colleagues reported the differentiation of adult human spermatogonia up to presumptive spermatocytes in cellular aggregates within collagen gel.^52^ Collagen sponges were later used to provide spatial environment allowing structural reorganization of prepubertal rat testicular cells.^50^ However, only the first steps of tubulogenesis could be observed. Because the mitotic activity of Sertoli cells is crucial for the elongation of the cords,^53^ mostly prepubertal rodent testicular cells have been used to establish tubulogenesis in‐vitro. But, for ethical reasons, immature testicular cells are difficult to obtain from human. However, recent findings in rodent studies using collagen matrices suggest that adult testicular cells could be an alternative source of proliferating Sertoli cells.^49^, ^51^ It was hypothesized that due to their undifferentiated status, neonatal testicular cells adapted easier to culture conditions. However, adult mouse Sertoli cells continued to proliferate within collagen.^49^ The authors further reported that laminin around Sertoli cell aggregates regulated the alignment of the cells. Interestingly, Kulibin & Malolina^51^ identified two distinct populations of adult mouse Sertoli cells, with different location in‐vivo. Collagen‐embedded Sertoli cells originating from the rete testis maintained their mitotic activity and ability to form cord‐like structures in‐vitro. Likewise, Mäkelä et al^54^ demonstrated that adult mouse Sertoli cells in seminiferous tubule cultures maintain the capacity to proliferate through a process of dediferentiation.

Artificial 3D scaffolds, such as soft agarose,^6^, ^55^, ^56^, ^57^ methylcellulose,^6^ poly(D,L‐lactic‐co‐glycolic acid),^58^ and polydimethylsiloxane nanotubes,^59^ have also been used to culture rodent testicular cells. Promising results have been obtained by Stukenborg et al,^6^ who were the first to report the successful generation of elongated spermatids in mouse testicular cell aggregates in soft agarose and methylcellulose. The former continued to be used to generate elongated spermatozoa in mouse,^55^ but also pachytene spermatocytes in rat testicular cell aggregates.^56^ Notably, agarose is not cell‐interactive and might be responsible for the low efficiency of tubulogenesis and spermatogenesis.^6^, ^55^, ^56^ Interestingly, germ cells outside of de‐novo formed SC aggregates died, presumably because of lack of support.^56^

The lack of reorganization of testicular cells within scaffold‐based culture systems questions the necessity of a scaffold to recreate tubulogenesis in‐vitro. For instance, de‐novo formation of seminiferous tubule‐like structures supporting germ cell differentiation has been reported without scaffold.^60^ Indeed, in their study, tubulogenesis relied solely on the self‐reorganization potential of cultured neonatal mouse testicular cell aggregates during two weeks on agarose gel at the gas‐liquid interphase. Although spermatogonia progressed up to meiosis, the lack of a circulatory system might have hampered germ cells to differentiate completely.^60^ In rotation culture, neonatal rat testicular cells were able to reorganize into tubule‐like structures, whereas adult testicular cells regained this capacity only when deprived of germ cells.^61^ Pendergraft et al^18^ cultured adult SSCs and immortalized Sertoli and Leydig cells in a hanging drop of medium supplemented with human testicular ECM. The 23‐day‐long culture period did not result in the characteristic reorganization of the testicular cells into tubule‐like structures either. However, elongated spermatids, high cell viability, and steroidogenic activity were reported in the testicular organoids. Phenotypical differences between immortalized and primary cells may have influenced the ability of the cultured cells to reorganize and promote IVS.^18^, ^62^ Interestingly, the authors used the developed human testicular organoids to study the reprotoxicity of the Zika virus.^63^ Similarly, neonatal mouse testicular organoids formed in microwell plates supplemented with Matrigel^®^ were used to test phthalates exposition to testicular function.^19^ Although the testicular organoids had a testis‐specific structure and expressed tight junctions, the tubular and interstitial compartments were reversed. Moreover, prepubertal human testicular cells could reorganize into organoids without the supplementation of Matrigel^®^.

Altogether, testicular cells could reorganize faster and show better organization in 3D matrices, while prepubertal testicular cells could also reorganize in a scaffold‐free environment. In‐vivo, differentiation of Sertoli cells occurs until puberty, leading to functional and structural changes that confer the cells the ability to support spermatogenesis. While the mitotic activity of Sertoli cells decreases during maturation, the expression of differentiation markers, such as transferrin, androgen‐binding protein, androgen‐receptors, and follicle stimulating hormone‐receptors, increases. Interestingly, recent findings suggest that adult Sertoli cells can reacquire mitotic activity under appropriate culture conditions.^49^, ^51^, ^54^ In‐vivo, adult Sertoli cells in cords reorient themselves into elongated polarized cells at the basement membrane and develop a blood‐testis barrier. Finally, they secrete fluid to create a lumen and become seminiferous tubules (Figure 1D). The blood‐testis barrier consists of a network of cellular junctions that restricts the flow of factors to the differentiating germ cells, thereby dividing the tubules into two distinct microenvironments. In‐vitro, some aspects of Sertoli cell differentiation could be observed in 3D culture. However, in most studies, Sertoli cells did not mature further than cord‐like structures.^33^, ^35^, ^42^, ^54^, ^59^ In general, Sertoli cells remained partially differentiated, which may have hindered meiotic progression of germ cells.^30^, ^56^, ^59^ Only when in‐vitro derived testicular cell aggregates were grafted under the skin of immunodeficient mice, more advanced morphological changes could take place.^30^ The presence of microvessels and other still unknown factors that are lacking in‐vitro is hypothesized to account for the better reorganizing properties of the grafts.^64^, ^65^

### 3.3 Novel 3D strategies for testicular tubulogenesis

The nanostructure and composition of the matrix influence the physiological and morphological behavior of the cultured cells. Yet, 2D and 3D cultures only allow initial steps of tubulogenesis. Reorganization of testicular cells into hollow tubules of mature Sertoli cells requires novel culture methodologies. For therapeutic uses, artificial scaffolds and/or defined recombinant human ECM proteins should be used. However, artificial scaffolds often lack or have limited biocompatibility, while the usage of synthetic biological scaffolds still results in highly unorganized testicular cells. Because the composition of ECM is tissue‐specific, decellularized testicular matrix (DTM) was considered as an optimal scaffold for recellularization with testicular cells. We were the first to derive and develop natural cytocompatible scaffolds from human testis.^66^ Agitating testicular fragments of 1 cm^3^ for 24 h in 1% sodium dodecyl sulfate detergent was more effective in decellularizing and removing DNA from the tissue while maintaining important ECM proteins compared to treatment in 1% Triton X‐100.^66^ Following recellularization of these scaffolds with adult or pubertal human testicular cells at the gas‐liquid interphase, testicular somatic and germ cells attached to the scaffold and created 3D mini‐testicular tissues referred to as testicular organoids.^16^ The testicular organoids were composed of proliferative spermatogonia and functional niche cells secreting testosterone and inhibin B for at least four weeks. However, complete differentiation of germ cells was not expected because the testicular organoids did not show the critical testicular compartmentalization. Remarkably, the morphology and functionality of testicular cells grown on DTM were similar to DTM‐free conditions. Recently, Vermeulen et al^67^ compared several decellularization protocols for prepubertal porcine testicular fragments. Following recellularization of the different scaffolds with adult human Sertoli cells, 0.01% sodium dodecyl sulfate + 1% Triton X‐100 and 0.05% trypsin + 0.02% ethylenediaminetetraacetic + 3% Triton X‐100 showed the most promising results in terms of attachment, proliferation, and functionality of Sertoli cells within newly formed cellular aggregates. Another study fabricated testis‐derived scaffolds by incubating ram testicular tissue in sodium dodecyl sulfate + Triton X‐100 for 48h, followed by acidification in acid peptin solution and freeze‐drying cycles.^20^ Although tubular‐like structures were not observed following inoculation of the scaffolds with neonatal mouse testicular cells, post‐meiotic cells could be generated.

The lack of a circulatory system might have hampered germ cells to differentiate completely in tubule‐like structures as mentioned before.^60^ Recently, organotypic culture of mouse testes in microfluidic devices containing a circulatory system has shown increased efficiency and duration of spermatogenesis.^11^, ^68^ Hosting testicular organoids in microfluidic devices might thus improve their functionality. Alternatively, reducing the size of the sample might also increase the availability of shortcoming nutrients and oxygen.^69^ In a study from our group, a mix of juvenile mouse interstitial cells and alginate was printed in a macroporous scaffold and neonatal tubular cells were seeded into the scaffold pores. In parallel, all testicular cell populations were cultured on cell‐free scaffolds. Although tubules were not formed in‐vitro, both approaches resulted in the formation of small‐sized cellular aggregates and completion of spermatogenesis.^8^

## 4 IMPACT OF MEDIUM COMPOSITION ON TUBULOGENESIS IN‐VITRO

In this section, the various medium compositions that have been used in the past to study testicular tubulogenesis in‐vitro starting from testicular cell suspensions will be summarized. A shift from undefined serum‐containing media toward defined serum‐free media has been observed. Several factors have been reported to stimulate tubulogenesis, SSC propagation, and spermatogenesis in‐vitro.

### 4.1 Basal medium

Since the first studies on testicular tubulogenesis in‐vitro, culture conditions have improved considerably in order to ensure the growth and maintenance of testicular cells. To date, studies of testicular tubulogenesis have mostly employed traditional basal media, such as Eagle's minimal essential medium (MEM)^23^, ^25^, ^26^, ^27^, ^37^, ^44^, ^56^, ^61^ and its variations α‐MEM,^8^, ^17^, ^20^, ^60^ α‐MEM/F12,^51^ Dulbecco's modified MEM (DMEM),^21^, ^22^, ^24^, ^28^, ^30^, ^31^, ^32^, ^33^, ^35^, ^38^, ^39^, ^50^, ^56^, ^59^ KnockOut DMEM,^8^ and DMEM/F12.^6^, ^19^, ^29^, ^40^, ^41^, ^42^, ^52^, ^54^, ^56^, ^57^, ^58^, ^67^ In contrast, the use of Roswell Park Memorial Institute (RPMI) 1640^49^, ^55^ and StemPro‐34‐based media^18^ has rarely been reported. Because of their scarcity and tendency for apoptosis in culture, SSCs should ideally be propagated in‐vitro prior to IVS. StemPro‐34‐based media have extensively been used for this purpose in mice.^70^ Recently, Kojima et al^69^ identified insulin as the factor in StemPro‐34‐based media responsible for testicular somatic cell growth in neonatal mouse organotypic culture. The use of DMEM/F12‐based medium resulted in a reduction of human testicular somatic cell overgrowth and may thus improve germ cell survival.^71^

### 4.2 Serum

Early 2D cultures of primary testicular cells typically used medium supplemented with serum.^21^, ^22^, ^25^, ^26^, ^27^, ^29^ However, none of these studies reported the successful reorganization of primary testicular cells into tubule‐like structures. The morphology of Sertoli cells did not change in culture with the addition of 2.5% fetal calf serum (FCS).^23^ Nevertheless, the addition of 10% FCS to immortalized Sertoli cells resulted in the transition from flat cords to tubular‐like structures.^24^ The authors suggested that hepatocyte growth factor, found in FCS, was responsible for the observed morphogenic effects. Because SV40 large T antigen was used to immortalize the Sertoli cells, the possibility exist that the oncogene also targeted pathways for cellular differentiation. Interestingly, in rat cord‐like structures cultured in Matrigel^®^, lumen formation could be observed in the absence of FCS.^30^ Others observed enrichment of Sertoli cells in serum‐free conditions.^28^, ^30^, ^41^ The authors explained this by the overgrowth of PTMCs when serum was added to the medium.^28^, ^30^, ^41^ Indeed, Legendre et al^41^ demonstrated that 10% FCS overstimulated PTMC proliferation. On the other hand, 10% FCS has successfully been used by Lee et al^40^, ^58^ who reported the generation of round rat spermatids in cellular aggregates in a mixture of Matrigel^®^ and collagen gel and in collagen gel alone, and presumptive elongated rat spermatids in aggregates in macroporous poly(D,L‐lactic‐co‐glycolic acid)‐based scaffolds. Using human adult testicular cells, the same authors reported the generation of elongated spermatids in collagen gel following 12 days of culture.^52^ Noteworthy, 30% FCS was used to generate testicular organoids from immortalized Sertoli cells and Leydig cells supporting progression of diploid to haploid cells.^18^ Aside from the immortalized cell line, starting material collected from adult men should be processed adequately because it can be contaminated with residual differentiating germ cells.

The age‐related changes in the concentration of serum proteins in addition to the batch variability make serum inappropriate to speculate about the mechanisms and factors driving testicular tubulogenesis and spermatogenesis in‐vitro. Undefined factors within serum may also have hampered these processes. As such, for further improvements of culture media, serum was replaced by the more reliable KSR supplement, commonly used to culture undifferentiated embryonic stem cells.^9^, ^72^ KSR consists of lipid‐rich albumin (AlbuMAX), amino acids, vitamins, transferrin, antioxidants, insulin, and trace elements.^72^ α‐MEM supplemented with KSR was previously found to allow IVS in neonatal mouse testicular organotypic cultures.^9^ The authors ascribed the effects of KSR to AlbuMAX,^9^, ^69^ which are believed to be exerted through the binding of albumin to other molecules.^73^ In selected studies starting from testicular cell suspensions, KSR has successfully been used for the generation of meiotic^49^ and post‐meiotic germ cells in mice,^20^, ^60^ meiotic initiation of rat spermatogonia,^42^ and maintenance of the mitotic activity of spermatogonia in rat^17^ and humans.^16^ However, in a study by von Kopylow et al,^32^ human spermatogonia were found only in low quantities in cord‐like structures following three months of culture in 2D uncoated dishes in DMEM supplemented with 15% KSR and growth factors (10 ng/mL insulin growth factor, 40 ng/mL embryonic growth factor, and 20 ng/mL fibroblast growth factor 1, 2, and 9). Noteworthy, the absence of a physiological 3D microenvironment may have accounted for this observation. The addition of 10% KSR to RPMI‐1640 promoted the formation of tubule‐like structures with formation of a blood‐testis barrier from dissociated neonatal rat testicular cells within collagen matrix.^49^ Within the structures, mature Sertoli cells reoriented to engulf germ cells and promoted germ cell proliferation and differentiation. Absence of KSR resulted in differences in behavior and morphology of Sertoli cells and PTMCs, and decreased aggregation of Sertoli cells.^49^ Recently, culture systems with a Matrigel^®^ overlay got improved by incorporating KSR, which promoted testicular cell survival and proliferation and meiotic induction of neonatal rat spermatogonia.^42^ In addition, most studies reporting the creation of testicular organoids incorporated 10% KSR in the culture medium.^8^, ^16^, ^17^, ^20^, ^60^ However, control conditions without KSR were not performed. The optimal concentration of KSR is believed to be around 10% because higher concentrations did not improve germ cell differentiation and testosterone production of mouse testicular cells in organotypic culture.^74^

### 4.3 Gonadotrophins/androgens

Follicle stimulating hormone (FSH) and luteinizing hormone (LH) are gonadotrophins secreted by the pituitary in response to gonadotrophin releasing hormone from the hypothalamus. Gonadotrophins have a central role in testicular development and function. While LH is responsible for testosterone production by the Leydig cells,^75^ FSH is a known driver of Sertoli cell proliferation after birth until they are fully mature at the onset of spermatogenesis during puberty. Inhibin B secretion by Sertoli cells will in turn modulate pituitary FSH secretion through negative feedback.^76^, ^77^ In response to FSH, mature Sertoli cells also support spermatogenesis through direct communication with germ cells. Moreover, the testicular somatic compartment provides germ cells with a paracrine milieu of Sertoli cell‐secreted growth factors such as stem cell factor (SCF), transforming growth factor (TGF)‐β, fibroblast growth factor (FGF), glial‐cell line‐derived neurotrophic factor (GDNF), androgen‐binding protein, and transferrin. The high affinity of androgen‐binding proteins for androgens contributes to the high intra‐testicular concentrations of testosterone, necessary for meiotic entry.^78^, ^79^ Elevated secretion of androgen‐binding protein was previously shown in co‐cultures of neonatal rat Sertoli cells and PTMCs in MEM supplemented with 100 ng/mL FSH.^25^ Moreover, 10 ng/mL FSH was shown to increase mRNA levels of GDNF, an import factor for SSC self‐renewal.^54^ Testosterone was incorporated in culture of rat^33^, ^35^, ^40^, ^41^, ^58^ and human^52^ testicular cells, while human chorionic gonadotrophin (hCG) was used as a surrogate for LH in culture of rat,^50^, ^56^ mouse,^6^, ^20^ and human^16^ testicular cells. However, the effects of testosterone or hCG have not been further characterized. Interestingly, Reda et al^56^ highlighted the role of glutamine in steroidogenesis of neonatal rat testicular cells in soft agarose. Glutamine,^18^, ^24^, ^32^, ^33^, ^35^, ^67^ and its more stable alternative formulation Glutamax^8^, ^16^, ^50^ have also been included in the culture medium by other researchers, but the effects have not been further characterized. Cord‐like structures could be obtained from neonatal rat testicular cells in Matrigel^®^ in serum‐free defined DMEM supplemented with 100 ng/mL FSH and 10^−8^ M testosterone.^33^, ^35^ However, the authors did not further characterize the effects of the gonadotrophins on testicular tubulogenesis. In another study, 200 ng/mL FSH was shown to stimulate Sertoli cell reaggregation on laminin.^28^ In addition, mound formation could only be observed in the presence of FSH.^28^ On the contrary, Gassei et al^38^ suggested that Sertoli cell aggregation was FSH‐independent. Neither 10‐100 ng/mL FSH, nor anti‐FSH receptor antibodies (1:100‐1:1000) disturbed Sertoli cell aggregation. Despite these observations, it should be noted that Sertoli cells did not proliferate in this culture setup. Therefore, these results should be interpreted carefully. Schlatt et al^28^ further showed that contact‐inhibition decreased the mitotic activity of densely packed Sertoli cells at high cell densities, an event also observed by Pan et al^59^ and originally described by Steinberger & Steinberger^80^ in cultured rat Sertoli cells. Proliferation could partially be reinitiated through the addition of 200 ng/mL FSH in the culture medium.^28^ Accordingly, testicular cell densities and ratios should be monitored to prevent somatic cell outgrowth, a major obstacle for SSC culture.

The crucial role of gonadotrophins in germ cell differentiation was highlighted by Stukenborg et al.^6^ Only when DMEM/F12 was supplemented with physiological concentrations of 5 IU/L FSH and hCG, neonatal mouse testicular cells in soft agarose and methylcellulose could differentiate elongated spermatids.^6^ Although the differentiation efficiency to haploid cells was low, the number of tetraploid cells (representing meiotic primary spermatocytes) increased in cultures with gonadotrophin supplementation. Interestingly, high serum levels of FSH (>12 IU/L) correlated with poor differentiation of human germ cells in‐vitro, while levels of 1‐6 IU/L have been associated with a good differentiation potential.^52^ In agreement with previous work on the steroidogenic and anti‐apoptotic effects of hCG supplementation,^81^, ^82^ 5 IU/L hCG drove testosterone production in Leydig cells and enhanced germ cell survival.^6^ Comparable results were obtained by Reda et al^56^ by culturing neonatal rat testicular cells in soft agarose in a similar medium composition. Nonetheless, when neonatal rat testicular cells were cultured in collagen sponges using the same medium composition, the steroidogenic and anti‐apoptotic effects of gonadotrophin supplementation could not be observed.^50^ A possible explanation for this observation is the inefficient biodegradability of the collagen sponges, or their lack of bioactivity. Stimulation of adult human and neonatal mouse testicular cells on DTM with gonadotrophins (5 IU/L FSH and hCG) did not further increase inhibin B and testosterone secretion by Sertoli and Leydig cells, respectively.^16^, ^20^ Baert et al^16^ reasoned that the age of the adult human donors could be the cause for the lack of response of the testicular cells. Alternatively, since control cultures (also showing inhibin B and testosterone secretion) included KSR or Glutamax, and since these factors have known gonadotrophin‐like effects,^56^ the addition of gonadotrophins might have been superfluous. Noteworthy, spermatids have been derived from in‐vitro systems of unorganized testicular cells in the absence of gonadotrophins, but supplemented with FCS^55^ or KSR.^8^, ^32^ It therefore remains difficult to evaluate the need of gonadotrophins in cultures studying testicular development, especially in cultures supplemented with undefined or lipid‐rich serum.^16^, ^18^, ^20^, ^40^, ^41^, ^52^, ^54^, ^58^

### 4.4 TGFβ‐superfamily

The TGFß‐superfamily contains a large group of proteins controlling testicular development such as activin A and GDNF. Activin A is a key regulator of Sertoli cell proliferation during embryogenesis and early postnatal life.^83^, ^84^ Its production decreases during puberty (when Sertoli cells differentiate) through the antagonizing effects of follistatin and FSH‐induced inhibin B. Activin A directly contributes to the establishment of Sertoli cell numbers, thereby determining the testis size and daily spermatozoa production.^85^, ^86^ Interestingly, it was previously reported that non‐proliferative adult Sertoli cells treated with 50 ng/mL activin A could dedifferentiate to a prepubertal phenotype exhibiting the ability to proliferate.^87^ The regulatory network of activin A, follistatin, and FSH may also regulate spermatogenesis. Indeed, activin A treatment reduced the expression of the germ cell differentiation marker KIT proto‐oncogene receptor tyrosine kinase (*KIT*) transcript levels in organotypic hanging drop cultures of human testis cancer samples.^88^ With the roles of activin and FSH on Sertoli cell proliferation and tubulogenesis in mind, rat Sertoli cells in 3D Matrigel^®^ surprisingly did not lose the ability to aggregate when treated with the activin antagonists follistatin (50‐500 ng/mL) or FSH (10‐100 ng/mL).^38^ The authors deduced that Sertoli cell aggregation was not an activin‐specific event, but could be the result of combined effects of different factors.

The role of GDNF as the most important regulator of SSC survival and self‐renewal is well established.^89^, ^90^ GDNF is an important medium component for murine and human SSC propagation culture systems.^70^, ^90^, ^91^ The secretion of GDNF in Sertoli cells is mostly FSH‐dependent.^92^ Additionally, retinoic acid (RA) treatment was shown to downregulate GDNF, further supporting the role of GDNF in inhibiting differentiation and maintaining the undifferentiated state of SSCs.^93^ The culture medium used to generate organoids from immortalized human testicular cells was similar to the one used for mouse SSC propagation, comprising 10 ng/mL GDNF.^18^, ^63^ Ten ng/mL GDNF was also added to 10% KSR to stimulate SSC self‐renewal in tubule‐like structures, a culture setup able to support the initial steps of meiosis in mouse.^60^ In another study, 100 ng/mL GDNF was combined with a cocktail of growth factors (20 ng/mL FGF1, FGF2 and FGF9, 10 ng/mL insulin growth factor and 40 ng/mL epidermal growth factor) to generate cord‐like structures from human testicular cells in 2D culture.^32^ In their 2D culture setup, germ cells could not be maintained and some somatic cells dedifferentiated. Nevertheless, the tubulogenesis‐inducing effects of GDNF were not evaluated in these studies. Moreover, recent studies have shown tubulogenesis without GNDF supplementation.^17^, ^30^, ^39^, ^41^, ^49^, ^51^, ^61^

### 4.5 Other growth factors

Male sex differentiation starts with the Y‐chromosome‐specific expression of sex‐determining region Y (*Sry*) in the fetal indifferent bipotential gonads through its main downstream effector, the transcription factor SOX9.^94^ FGF9 is an established downstream effector of SRY/SOX9 in pre‐Sertoli cells of the developing fetal testis where it is responsible for differentiation of pre‐Sertoli cells to Sertoli cells. Factors secreted by Sertoli cells will subsequently drive the commitment of precursor somatic testicular niche cells toward male sex differentiation.^95^ FGF9 induces proliferation of Sertoli cells and also suppresses meiotic entry of germ cells by antagonizing retinoic acid 8 (*Stra8*) expression.^94^ The meiotic block of germ cells during fetal life occurs through the upregulation of NANOS2, referred to as the meiotic gatekeeper.^96^ It is an RNA‐binding protein that silences the RA/SCF/KIT axis that is essential for meiotic entry of germ cells. Nonetheless, experiments focusing on the role of FGFs in testicular tubulogenesis are scarce.^18^, ^22^, ^24^, ^29^, ^32^ Consistent with the previous work from Colvin et al^94^ in‐vivo, 10 ng/mL FGF9 enhanced proliferation and aggregation of fetal mouse Sertoli cells into cord‐like structures on Matrigel^®^.^22^ Moreover, in the presence of 20 ng/mL FGF9, adult human testicular cells reorganized into cord‐like structures in 2D culture.^32^ Few studies have focused on FGF2, a key regulator of in‐vitro SSC propagation. In co‐culture of fetal and prepubertal rat Sertoli cells and SSCs, it was shown that 4‐10 ng/mL FGF2 acted as a testicular morphogen through survival and mitogenic actions.^97^ However, it was recently reported that 10 μg FGF2/adult mouse testis induces differentiation of spermatogonia.^98^ Although the number of spermatogonia increased under influence of FGF2, these cells developed into a differentiation‐prone subset expressing the RA receptor RARγ. Moreover, FGF2 signaling was shown to suppress both GDNF production and degradation of RA in‐vivo*.* 98 Fifty ng/mL FGF9 and FGF2 were found to promote reorganization of prepubertal rat Sertoli cells into aggregates. The FGF2‐treated aggregates were bigger than the FGF9‐treated ones. This difference could be attributed to the survival and mitogenic actions of FGF2 on PTMCs. The authors further suggested that FGFs take part into the remodeling of the basement membrane of the tubules by regulating the expression of proteinases.^29^

Originally identified for their nerve growth‐stimulating activity, several neurotrophins have been identified in the developing testis of rodents^99^, ^100^, ^101^, ^102^ and humans.^103^ These neurotrophins comprise brain‐derived neurotrophic factor (BDNF), nerve growth factor (NGF), and neurotrophin (NTF) 3. The actions of NGF, BDNF, and NTF3 are mediated through the neurotrophin‐specific tyrosine kinase receptors (NTRK) 1, NTRK2, and NTRK3, respectively. It was suggested that neurotrophins could have a role in testicular development.^101^ When treated with high doses of the NTRK inhibitor K252a (5‐10 nM), Sertoli cells did not aggregate.^38^ A similar dose‐dependent effect on Sertoli cells was shown with the NTRK1‐specific antagonist AG879 (10‐20 μM).^38^ However, treatment of the testicular cells with NTF3 (10‐100 ng/mL) did not stimulate aggregation, nor rescued the inhibitory effects of the NTRK antagonists.^38^ The disturbance of K252a on Sertoli cell aggregation was also observed in humans, where relatively high doses (500 nM and higher) prevented cluster formation by inhibiting the protrusion of PTMCs.^32^

### 4.6 Vitamin A

In order to improve the meiotic process that has long been proven difficult in IVS,^104^ the supplementation of culture media with retinoids (retinol and RA) has been considered. RA is the biological active metabolite produced from retinol. RA plays a central role in meiotic entry of germ cells in both rodents and humans.^105^, ^106^ The actions of RA in the testis are mediated through the RA receptors, of which RARα, RARß, and RARγ are expressed on Sertoli cells, round spermatids, and undifferentiated spermatogonia, respectively.^107^ RA directly induces the transition from undifferentiated KIT^‐^ to differentiating KIT^+^ spermatogonia by upregulating STRA8. Indirectly, RA also does so by stimulating the secretion of bone morphogenetic protein 4 and SCF by Sertoli cells and by downregulating GDNF.^93^ This will, in turn, induce the synthesis of meiotic markers in germ cells, such as synaptonemal complex protein 3 and meiosis‐specific recombinase.^93^

In initial culture conditions, serum acted as a source of RA.^108^ Serum‐free culture media were supplemented with 50 ng/mL vitamin A for the culturing of immature rat testicular cells in Matrigel^®^.^33^, ^35^ Later, its in‐vitro role shifted to promoting differentiation of germ cells.^17^, ^18^, ^19^, ^20^, ^40^, ^41^, ^52^, ^58^ Indeed, Lee et al generated round spermatids in‐vitro in rat and presumptive elongated spermatids in human using a medium supplemented with a combination of RA and retinol.^40^, ^52^ Legendre et al^41^ observed round spermatids in their in‐vitro model with little adjustments to the medium composition used by Lee et al.^40^, ^52^ RA has later been used at different concentrations to generate post‐meiotic germ cells in testicular organoids made from adult human and neonatal mouse testicular cells.^18^, ^20^ Although Topraggaleh et al observed a significantly higher expression of post‐meiotic genes following administration of 1 μM RA, expression of *Stra8* did not change significantly in culture, nor did synaptonemal complex protein 3.^20^ Using a three‐layer gradient system of Matrigel^®^, Alves‐Lopes et al^17^ investigated the role of RA in IVS. Through treatment of the testicular organoids with 10 nM‐10 μM RA and the RA antagonist ER 50 981, they concluded that RA improved germ cell counts (12%) in 21 days culture compared with controls (7%). However, when a higher concentration of RA (10 μM) was used, this effect was countered. Noteworthy, it was recently demonstrated in neonatal mouse organotypic cultures that 10 μM retinol was more effective than RA in inducing seminiferous tubule growth and meiosis.^109^ Similarly, the effects of RA on germ cells in human testicular organoids were weaker compared to the effects on germ cells in 2D culture.^19^ These studies support the idea that reorganized PTMCs around the seminiferous tubules may act as RA‐degrading barrier that inhibits RA actions in the tubules through cytochrome P450 hydroxylase enzymes.^107^

## 5 CONCLUSION

Most IVS studies using testicular cell suspensions have focused on obtaining post‐meiotic germ cells without paying attention to also improve the reestablishment of the testicular architecture. However, the testicular cell organization is pivotal in achieving spermatogenesis in‐vitro. With this review, we summarized and compared studies aiming to recreate an adequate in‐vitro environment for testicular cells in order to mimic testicular tubule formation and germ cell differentiation in‐vitro. The testicular organoid concept is emerging in tissue engineering and might allow the creation of a functional human testicular surrogate from isolated testicular cells, especially with the emergence of 3D bioprinting. The regulation of testicular tubulogenesis in‐vitro remains poorly understood as tubular‐like structures were rarely able to support IVS. Moreover, most of the selected studies have been conducted in rodents. Although rodent IVS systems can provide much insight into human spermatogenesis, it is crucial to develop systems that recapitulate the actual human spermatogenesis as this process shows differences with rodents. Given the long cycle of human spermatogenesis, it will be necessary to maintain long‐term testicular cell cultures, while providing signals important for germ cell differentiation. Taking into account the different steps in testis development and germ cell differentiation (mitosis, meiosis, and spermiogenesis), sequential culture media might need to be developed in order to promote tubulogenesis and germ cell differentiation. The results suggest prepubertal testicular cells possess a self‐assembly potential that has to be taken full advantage of by improving the medium composition. Nonetheless, if adult testicular cells cannot be induced to dedifferentiate into morphogenic cells, 3D bioprinting technology might be required because it gives control over cell deposition and scaffold design. This concern is particularly relevant for humans as prepubertal material is scarce. From the medium ingredients, KSR has been proven critical for the reorganization and in‐vitro maturation of rodent testicular cells. However, the exact factor within KSR responsible for this has yet to be defined. Although KSR was also successful in maintaining human germ cells in testicular organoids, it remains to be tested whether this is sufficient to induce complete differentiation of human SSCs. Possibly, other combinations of factors are needed with respect to tubulogenesis. However, because of the rich medium compositions used in selected studies, it is difficult to make definite conclusions. Recent findings suggest that FGFs and neurotrophins require more research focus. Furthermore, vitamin A derivates may be used to improve the efficiency of spermatogenesis. Other cell types and factors which have not been studied in included studies, for example, endothelial cells, BMP’s, and SCF, deserve more attention.

## CONFLICT OF INTEREST

The authors declare there are no conflicts of interest.

## AUTHORS’ CONTRIBUTIONS

GR contributed to conception and design, literature search, and manuscript writing; GR, Y.B, and EG contributed to manuscript revision and final approval of the manuscript.
