# Supplementary material for: In‐vitro spermatogenesis through testis modelling: Toward the generation of testicular organoids
Source: Andrology. 2020 Jan 9;8(4):879–91. doi: 10.1111/andr.12741 (PMC7496450; doi:10.1111/andr.12741)
Supplement: Supplementary file 1 [file ANDR-8-879-s001.pdf]

Supplementary table 1 Overview of different combinations of 2D culture methodology and signaling factors on best outcomes per study

AUTHORS	DATE	START	CULTURE METHODOLOGY	TIME IN CULTURE	CELL REORGANIZATION	SPERMATOGENESIS	BASAL MEDIUM	SERUM	GONADOTROPHINS	TGFβ-SUPERFAMILY	GROWTH FACTORS	VITAMIN A	OTHER INGREDIENTS
**Tung and Fritz**	**1980**	20 dpp rat SCs and PTMCs	Non-coated	90 days	Mounds	N/A	MEM	10% FCS	100 ng/ml FSH	-	-	-	-
**Tung and Fritz**	**1984**	20 dpp rat SC	ST biomatrix	3 weeks	Cord-like structures	N/A	MEM	-	-	-	-	-	-
**Tung and Fritz**	**1986**	20 dpp rat SCs and PTMCs	Collagen I, IV, LN or fibronectin	16 days	Cord-like structures	N/A	MEM	10% FCS	-	-	-	-	-
**Tung and Fritz**	**1987**	20 dpp rat SCs and PTMCs	Non-coated	18 days	Cord-like structures	N/A	MEM	10% FCS	-	-	-	-	-
**Schlatt et al.**	**1996**	7 dpp rat SCs and PMTCs	LN	7 days	Mounds	N/A	DMEM	-	200 ng/ml FSH	-	-	-	-
**Van der Wee and Hofmann**	**1999**	10 dpp immortalized mouse SCs	GFR-Matrigel	5 days	Tubule-like structures	N/A	DMEM	10% FCS	-	-	-	-	1 mM sodium pyruvate<br>2 mM glutamine<br>100 uM NEAA
**Mackay et al.**	**1999**	Embryonic mouse SCs	Matrigel	7 days	Cord-like structures	N/A	DMEM	10% FCS	-	-	-	-	-
**Willerton et al.**	**2004**	Embryonic mouse SCs	Matrigel	7 days	Cord-like structures	N/A	DMEM	10% FCS	-	-	10 ng/ml FGF9	-	-
**El Ramy et al.**	**2005**	20 dpp rat SCs and PTMCs	Non-coated	5 days	Cord-like structures	N/A	DMEM/F12	10% FCS	-	-	50 ng/ml FGF9<br>50 ng/ml FGF2<br>50 ng/ml PDGF	-	-
**Mincheva et al.**	**2018**	Adult human TCs	Glass	3 weeks	Cord-like structures	N/A	DMEM	-	-	-	-	-	1% NEAA
**Von Kopylow et al.**	**2018**	Adult human TCs	Non-coated	3 months	Cord-like structures	ES, SG loss	DMEM	15% KSR	-	100 ng/ml GDNF	40 ng/ml EGF<br>20 ng/ml FGF1<br>20 ng/ml FGF2<br>20 ng/ml FGF9<br>100 ng/ml GDNF<br>10 ng/ml IGF	-	0.1 mM 2-Mercaptoethanol<br>2 mM glutamine<br>0.1 mM NEAA

ddp; days postpartum; ES, elongates spermatids; GFR, growth factor reduced; LN, laminin; NEAA, non-essential amino-acids; PTMC, peritubular myoid cells; SC, Sertoli cells; SG, spermatogonia; ST, seminiferous tubule; TC, testicular cells
