# Supplementary material for: In‐vitro spermatogenesis through testis modelling: Toward the generation of testicular organoids
Source: Andrology. 2020 Jan 9;8(4):879–91. doi: 10.1111/andr.12741 (PMC7496450; doi:10.1111/andr.12741)
Supplement: Supplementary file 2 [file ANDR-8-879-s002.pdf]

Supplementary table 2 Overview of different combinations of 3D culture methodology and signaling factors on best outcomes per study

AUTHORS	DATE	START	CULTURE METHODOLOGY	TIME IN CULTURE	CELL REORGANIZATION	SPERMATOGENESIS	BASAL MEDIUM	SERUM	GONADOTROPHINS	TGFβ-SUPERFAMILY	GROWTH FACTORS	VITAMIN A	OTHER INGREDIENTS
**Zenzes and Engel**	**1981**	8, 10, 18, 25, 35, 40, 90 dpp rat TCs	Rotation culture	-	Tubule-like structures	N/A	MEM	-	-	-	-	-	-
**Hadley et al.**	**1985**	10 dpp rat Sertoli cells	Matrigel	5 weeks	Cord-like structures	PaS	DMEM	-	100 ng/ml FSH<br>10^{-8} M teststerone<br>10^{-8} M estradiol	-	10 ng/ml EGF<br>2 ug/ml insulin	50 ng/ml vitamin A	5 ug/ml transferrin<br>200 ng/ml vitamin E<br>10^{-9} M hydrocortisone<br>2 mM glutamine<br>5 ng/ml sodium selenate<br>1 mM sodium pyruvate<br>22 mM sodium lactate<br>3 mg/ml cytosine arabinoside<br>50 ng/ml vitamin C
**Hadley et al.**	**1990**	10 dpp rat SCs	Matrigel	3 weeks	Cord-like structures	N/A	DMEM	-	100 ng/mL FSH<br>10^{-8} M teststerone<br>10^{-8} M estradiol	-	10 ng/ml EGF<br>2 ug/ml insulin	50 ng/ml vitamin A	5 ug/ml transferrin<br>200 ng/ml vitamin E<br>10^{-9} M hydrocortisone<br>2 mM glutamine<br>5 ng/ml sodium selenate<br>1 mM sodium pyruvate<br>22 mM sodium lactate<br>3 mg/ml cytosine arabinoside<br>50 ng/ml vitamin C
**Yu et al.**	**2005**	5 dpp rat SCs and SG	Matrigel overlay	2 weeks	Cellular aggregates	N/A	MEM	-	-	-	-	-	0.1 mM NEAA<br>1 mM sodium pyruvate<br>3 mM sodium lactate<br>1% ITS
**Gassei et al.**	**2006**	7 dpp rat TCs	Matrigel	5 weeks	Tubule-like structures	N/A	DMEM	-	-	-	-	-	NEAA
**Lee et al.**	**2006**	18 dpp rat TCs	CGM	22 days	Cellular aggregates	RS	DMEM/F12	10% FCS	2.5 x 10^{-5} IU FSH<br>10^{-7} M testosterone	-	-	3.3 x 10^{-7} M RA<br>3.3 x 10^{-7} M retinol	10 mg/ml ITS<br>10^{-4} M vitamin C<br>10 ug/ml vitamin E<br>1 mM pyruvate
**Lee et al.**	**2007**	Adult human TCs	Collagen	12 days	Cellular aggregates	SpT	DMEM/F12	10% FCS	100 mIU FSH<br>10^{-7} M testosterone<br>10^{-7} M DHT	-	-	3.3 x 10^{-7} M RA<br>3.3 x 10^{-7} M retinol	10 mg/ml ITS<br>10^{-4} M vitamin C<br>10 ug/ml vitamin E<br>1 mM pyruvate
**Gassei et al.**	**2008**	7 dpp rat TCs	Matrigel	9 days	Cellular aggregates	N/A	DMEM	-	10 - 100 ng/ml FSH	-	-	-	1% NEAA<br>10 ng/ml - 100 ng/ml NTF3
**Stukenborg et al.**	**2008**	7-10 dpp mouse TCs	Soft agarose	15 days	Cellular aggregates	RS	DMEM/F12	-	-	-	-	-	-
**Stukenborg et al.**	**2009**	7-10 dpp mouse TCs	Soft agarose	31 days	Cellular aggregates	ES	DMEM/F12	-	5 IU/l hCG<br>5 IU/l FSH	-	-	-	-
**Stukenborg et al.**	**2009**	7-9 dpp mouse TCs	Methylcellulose	31 days	Cellular aggregates	ES	DMEM/F12	-	5 IU/l hCG<br>5 IU/l FSH	-	-	-	-
**yu et al.**	**2009**	5 dpp rat SCs and SG	Matrigel	7 days	Cellular aggregates	N/A	MEM	-	-	-	-	-	-
**Legendre et al.**	**2010**	18 dpp rat TCs	Matrigel, collagen I (bicameral chamber)	22 days	Tubule-like structures	RS	DMEM/F12	5% FCS	100 mIU FSH<br>10^{-7} M testosterone	-	-	3.3 x 10^{-7} M retinol	6.25 ug/ml ITS<br>10^{-4} M vitamin C<br>10 ug/ml vitamin E<br>1 mM pyruvate
**Gassei et al.**	**2010**	7 dpp rat TCs	Matrigel	9 days	Tubule-like structures	N/A	DMEM	-	-	-	-	-	1% NEAA

**Lee et al.**	**2011**	18 dpp, 30 dpp rat TCs	PLGA	18 days	Cellular aggregates	ES	DMEM/F12	10% FCS	100 mIU FSH<br>10^{-7} M testosterone	-	-	3.3×10^{-7} M RA<br>3.3×10^{-7} M retinol	10 mg/ml ITS<br>10^{-4} M vitamin C<br>10 ug/ml vitamin E<br>1 mM pyruvate
**Abu elhija et al.**	**2012**	7 dpp mouse TCs	SACS	4 weeks	Cellular aggregates	ES	RPMI-1640	20% FCS	-	-	-	-	-
**Yokonishi et al.**	**2013**	0.5-5.5 dpp mouse TCs	Cellular aggregates	61 days	Tubule-like structures	RS	Alpha-MEM	10% KSR	-	10 ng/ml GDNF	-	-	-
**Pan et al.**	**2013**	7 dpp rat TCs	Nanotubes (PDMS)	15 days	Cord-like structures	N/A	DMEM	-	-	-	-	-	-
**Mäkelä et al.**	**2014**	<10 dpp to 17 dpp mouse ST	Plastic	8 weeks	Cord-like structures	N/A	DMEM/F12	15% FCS	10 ng/ml FSH	10 ng/ml GDNF	-	-	Angiotensin II
**Zhang et al.**	**2014**	6 dpp mouse TCs	Collagen	3 weeks	Tubule-like structures	PaS	RPMI-1640	10% KSR	-	-	-	-	-
**Reuter et al.**	**2014**	7 dpp rat TCs	Collagen sponges	35 days	Cellular aggregates	None	DMEM	-	5 IU/l hCG<br>5 IU/l FSH	-	-	-	Glutamax
**Reda et al.**	**2014**	7 dpp rat TCs	SACS	31 days	Cellular aggregates	PaS	DMEM<br>DMEM/F12<br>F12<br>MEM	-	5 IU/l hCG<br>5 IU/l FSH	-	-	-	Glutamax
**Kulibin et al.**	**2016**	4-6 dpp, 8-12 dpp mouse SCs	Collagen	10 days	Tubule-like structures	N/A	Alpha-MEM/F12	5% FCS	-	-	-	-	-
**Pendergraft et al.**	**2017**	Adult human TCs (immortalized SC, Leydig cells)	Hanging drop, ultra-low attachment U-plates + 1 ug/ml ECM extract	23 days	Cellular aggregates	Haploid germ cells	StemPro-34	30% FCS	2.5×10^{-5} IU FSH	10 ng/ml GDNF	100 ng/mL SCF<br>10 ng/ml FGF2<br>20 ng/ml EGF<br>25 ug/ml insulin<br>10 ng/ml LIF	2 uM RA	100 ug/ml transferrin<br>60 uM putrescine<br>30 nM sodium selenite<br>6 mg/ml D-(+)-glucose<br>30 ug/ml pyruvic acid<br>1 ul/ml DL-lactic acid<br>5 mg/ml bovine albumin<br>2 mM glutamine<br>5×10^{-5} 2-mercaptoethanol<br>Vitamin solution<br>NEAA<br>10^{-4} M ascorbic acid<br>10 ug/ml d-biotin<br>30 ng/ml β-estradiol<br>60 ng/ml progesterone
**Alves-Lopes et al.**	**2017**	5-8 dpp, 20 dpp, 60 dpp TCs	Matrigel (3 LGS)	21 days	Tubule-like structures	Proliferative SG	Alpha-MEM	10% KSR	-	-	-	10 nM - 10 uM RA	10^{-7} M melatonin
**Zhang et al.**	**2017**	6 dpp rat TCs	Matrigel overlay	12 days	Cord-like structures	Meiotic initiation	DMEM/F12	10% KSR	-	-	-	-	-
**Baert et al.**	**2017**	Adult & adolescent human TCs	DTM (human)	1 month	Cellular aggregates	Proliferative SG	KODMEM	10% xenofree KSR	5 IU/l hCG<br>5 IU/l FSH	-	-	-	1x Glutamax
**Vermeulen et al.**	**2018**	Adult human SCs	DTM (porcine)	18 days	Cellular aggregates	None	DMEM/F12	5% FCS	-	-	-	-	15 mM HEPES<br>2.5 mM glutamine
**Topraggaleh et al.**	**2018**	3-5 dpp mouse TCs	DTM (ram)	30 days	Cellular aggregates	RS	Alpha-MEM	10% KSR	5 IU/l hCG<br>5 IU/l FSH	-	-	10^{-6} M RA	-
**Sakib et al.**	**2019**	8-10 dpp mouse TCs, 6 months & 5 year old human TCs	Microwell (+ 1:100 Matrigel)	7 days	Cellular aggregates (reverse polarity)	N/A	DMEM/F12	-	-	-	20 ng/ml EGF<br>10 ug/ml insulin	1 uM RA	5.5 ug/ml transferrin<br>6.7 ng/ml selenium
**Baert et al.**	**2019**	5-7 dpp mouse TCs, >7 dpp interstitial cells	3D printed Alginate-RGD scaffold	35 days	Cellular aggregates	ES	Alpha-MEM	10% KSR	-	-	10^{-7} M Melatonin	-	1x Glutamax

CGM, collagen gel-Matrigel; ddp; days postpartum; DHT, dihydrotestosterone; ES, elongates spermatids; GFR, growth factor reduced; LGS, layer-gradient system; LN, laminin; NEAA, non-essential amino-acids; PLGA, poly(D,L-lactic-co-glycolic acid); PaS, pachytene spermatocytes; PMDS, polydimethylsiloxane; PTMC, peritubular myoid cells; RS, round spermatids; SACS, soft-agarose culture system; SC, Sertoli cells; SG, spermatogonia; ST, seminiferous tubules; TC, testicular cells
